# Social Relationships in Older Adulthood and Links With Psychological and Physical Well-Being

**DOI:** 10.1093/geroni/igab046.817

**Published:** 2021-12-17

**Authors:** Emily Willroth, Patrick Hill

## Abstract

Positive social relationships are fundamental to psychological and physical well-being across the lifespan. This symposium showcases rigorous daily-diary and longitudinal investigations that (a) examine change in social relationships and loneliness in older adulthood, and (b) investigate links between social relationships and psychological and physical well-being outcomes in older adulthood. First, we present results from a coordinated analysis of three longitudinal studies demonstrating that loneliness tends to increase across the second half of life (Talk 1). Second, we share converging evidence that suggests positive social relationships tend to decline with age. In turn, these longitudinal changes in loneliness and social relationships predict later physical health outcomes (Talk 2). Together, these findings suggest that positive social relationships tend to decrease and loneliness tends to increase with age, resulting in physical health costs. In the second half of the symposium, we turn to research on how positive social relationships may promote psychological well-being, and in turn, better physical health in older adulthood. Using daily diary data, we demonstrate that on days when older adults report more positive social interactions, they also report feeling more sense of purpose (Talk 3). Finally, we show that higher sense of purpose and more positive change in sense of purpose in midlife prospectively predicts better physical health in older adulthood (Talk 4). Together, the research presented in this symposium reveals normative declines in social relationships in late life, while also highlighting the potential health benefits of increasing positive social relationships in older adulthood.

